# Psychiatric disorders and intellectual disability impact epilepsy care in adolescents: A nationwide registry study

**DOI:** 10.1002/epi4.70314

**Published:** 2026-08-01

**Authors:** Jesse Sobovitch, Colin Reilly, Johan Zelano, Rianne J. M. Goselink

**Affiliations:** ^1^ The Faculty of Medicine and Health Sciences Linköping University Linköping Sweden; ^2^ Paediatric Neurology Department, Queen Silvia Children's Hospital Sahlgrenska University Hospital, Member of the ERN EpiCARE Gothenburg Sweden; ^3^ Institute of Clinical Sciences, Sahlgrenska Academy University of Gothenburg Gothenburg Sweden; ^4^ Young Epilepsy Lingfield Surrey UK; ^5^ Department of Clinical Neuroscience, Institute of Neuroscience and Physiology, Sahlgrenska Academy University of Gothenburg Gothenburg Sweden; ^6^ Department of Neurology Sahlgrenska University Hospital, Member of the ERN EpiCARE Gothenburg Sweden; ^7^ Wallenberg Center for Molecular and Translational Medicine University of Gothenburg Gothenburg Sweden; ^8^ Department of Neurology Jönköping, Region Jönköping County Jönköping Sweden; ^9^ Department of Biomedical and Clinical Sciences Linköping University Linköping Sweden

**Keywords:** healthcare utilization, intellectual disability, psychiatric comorbidity, transition to adult care, young people with epilepsy

## Abstract

**Objective:**

To investigate the associations of intellectual disability (ID) and psychiatric comorbidities with healthcare utilization and mortality among adolescents with epilepsy.

**Method:**

A nationwide, population‐based observational study using the data from Swedish national patient registries was conducted. Individuals born during 1997–2001 with epilepsy were included and data on healthcare utilization, prescriptions, comorbidities, socioeconomic status and mortality were obtained during a 5‐year period (16–20 years of age). We examined associations of psychiatric disorders and ID with healthcare utilization and mortality using unadjusted and covariate‐adjusted models. A time‐lagged sensitivity analysis was conducted to address potential reverse causation.

**Results:**

Of the 2116 participants included in the study, 33% had specialist care contact for ID and 43% had contact for psychiatric disorders. Psychiatric disorders were, after adjustment for covariates, associated with higher odds of both epilepsy‐related and all‐cause emergency care and inpatient care. Having ID was associated with lower odds of epilepsy‐related and all‐cause emergency care, but higher mortality. Furthermore, several psychiatric disorders were linked to higher odds of all‐cause or epilepsy‐related inpatient care or all‐cause emergency care.

**Significance:**

Psychiatric comorbidities and ID are common in young people with epilepsy and were associated with higher healthcare utilization and mortality in the unadjusted analysis. When adjusting for covariates, psychiatric comorbidity was linked to increased care consumption, while having ID was linked to lower emergency care use but higher mortality. These findings indicate that comorbidities are clinically relevant in young persons with epilepsy and support the need for tailored, multidisciplinary follow‐up during this important life period.

**Plain Language Summary:**

Many adolescents with epilepsy also have mental health or learning difficulties, which may affect their need for healthcare. In this Swedish nationwide study, psychiatric conditions were linked to more emergency visits and unplanned hospital admissions, while intellectual disability was associated with fewer emergency visits but a higher risk of death. These findings highlight the need for tailored, coordinated support for young people with epilepsy during the transition to adult care.

**Key points**

- A nationwide registry study on the effects of comorbidity in 2116 adolescents with epilepsy.
- Forty‐three percent had specialist care contact for psychiatric diagnoses and 33% for intellectual disability.
- Psychiatric comorbidity was associated with more all‐cause and epilepsy‐related emergency visits and unplanned admissions.
- Intellectual disability was associated with fewer emergency visits but higher mortality.

## 1 INTRODUCTION

A substantial proportion of children with epilepsy continue to have epilepsy during adolescence and adulthood, requiring ongoing medical follow‐up and psychosocial support.^1^ Epilepsy in childhood is associated with adverse long‐term outcomes, including reduced quality of life, increased morbidity, and premature mortality, particularly among those with persistent seizures despite antiseizure medication (ASM) treatment.^2^, ^3^, ^4^ Adolescence is a particularly important period in this context, as it involves major physical, psychological, and social development alongside increasing responsibility for disease management and transfer from pediatric to adult healthcare services.^5^, ^6^

Adolescence is a well‐recognized period of vulnerability for young people with epilepsy.^5^, ^6^, ^7^ Poorly managed transition has been associated with loss of follow‐up, worsening seizure control, inadequate management of comorbidities, and poorer psychosocial outcomes, including increased risk of serious complications such as sudden unexpected death in epilepsy (SUDEP).^6^, ^7^ These challenges may be especially pronounced in children with epilepsy and neurological, psychological, or physical comorbidities as complex healthcare needs complicate transition to adulthood.^8^ Intellectual disability (ID) and psychiatric disorders are the most frequent comorbidities in people with epilepsy: around 30%–40% have ID, around 20% autism spectrum disorder, and 32% attention deficit hyperactivity disorder (ADHD).^9^, ^10^, ^11^

Among young people with epilepsy, those with intellectual disability (ID) represent a particularly vulnerable subgroup during adolescence.^6^, ^12^ In this subgroup, epilepsy is often linked to structural abnormalities or genetic syndromes, epilepsy may have an earlier onset, and may follow a more severe or treatment‐resistant course.^12^, ^13^, ^14^ Children with epilepsy and ID have complex healthcare needs, which extend far beyond seizure management. The burden of care may be further complicated by cognitive and communication difficulties, behavioral challenges, and multiple coexisting health conditions, all of which can make access to appropriate and continuous care more difficult.^12^, ^15^ Young people with both epilepsy and ID are therefore more likely to remain dependent on caregivers and to require coordinated, long‐term, multidisciplinary care during adolescence and adulthood.^12^, ^16^ These difficulties become more pronounced and tangible during adolescence as more healthcare participation and responsibility are expected.

Adolescents with epilepsy and ID face multiple transitions during adolescence, including transfers to various adult clinics that can fragment care, while their social, financial, and legal support systems also change.^6^, ^12^, ^16^, ^17^ This leads to an increased care burden on the caregivers as many adolescents and young adults continue to depend largely on family for their care organization.^12^, ^15^, ^16^ However, transition services addressing this important group remain limited, as existing programs are rarely tailored to young people with neurodevelopmental impairments or ongoing dependence on caregivers.^5^, ^7^, ^18^

Up to 60% of young persons with epilepsy have associated mental health difficulties or psychiatric comorbidity, including ADHD, autism spectrum disorder, anxiety, depression, and psychosis.^11^, ^19^, ^20^, ^21^, ^22^ These comorbidities may increase clinical complexity and influence seizure management, healthcare utilization, and overall quality of life.^19^, ^23^, ^24^, ^25^ Psychiatric comorbidity is associated with a reduced quality of life in children with epilepsy and worse mental health in caregivers.^26^ Additionally, psychiatric comorbidities may be associated with increased healthcare consumption in young persons with epilepsy.^25^

Although previous studies have demonstrated that epilepsy and psychiatric comorbidities and ID are associated with adverse outcomes,^23^, ^24^, ^25^, ^27^, ^28^ there is still limited population‐based evidence on how intellectual and psychiatric comorbidities influence healthcare utilization and mortality among adolescents with epilepsy during the transition to adult care. Improved knowledge in this area is important for identifying high‐risk subgroups, informing service planning, and guiding interventions aimed at improving continuity and quality of care.^6^, ^7^, ^18^ Therefore, this nationwide registry study aimed to investigate the influence of intellectual and psychiatric comorbidities on healthcare utilization and mortality among adolescents with epilepsy.

## 2 MATERIALS AND METHODS

### 2.1 Participants

A population‐based, retrospective cohort study utilizing registry data was performed. Data were collected on individuals born between January 1, 1997 and December 31, 2001 who fulfilled both of the following inclusion criteria:

- One or more specialist care contacts with epilepsy registered as the main diagnosis in the ICD‐10 (code G40) in the Swedish National Patient Register (NPR) during both their 18th year of life and their 16th or 17th year of life.
- One or more prescribed and dispensed ASM in the Drug Prescription register during the 18th year of life.

These criteria were chosen to identify young people with ongoing, treated epilepsy in late adolescence, rather than individuals with a remote or uncertain epilepsy diagnosis. Requiring epilepsy‐related specialist care contacts at two time points together with dispensed ASM during the 18th year was intended to increase the likelihood that the cohort represented “active” epilepsy at the time of transition from pediatric to adult care.^29^

For each individual, data were collected over five consecutive years, beginning with their 16th year of life. This corresponds to data collection spanning the calendar years 2013–2021. Data were collected from the Swedish National Board of Health and Welfare (Socialstyrelsen) and Statistics Sweden (SCB). A detailed description of the registries used is provided in an earlier publication.^5^ In short, the NPR contains data on inpatient care and specialist outpatient care in Sweden; the National Cause of Death Register provided data on deaths and cause of death; the Drug Prescription register contains data on dispensed prescribed medications. The longitudinal integrated database for health insurance and labor market studies (LISA) provided demographic and socioeconomic data. These registries have near‐complete national coverage and high accuracy, as the registries are compulsory.^30^

Diagnoses in the NPR were classified according to ICD‐10‐SE, the official Swedish adaptation of ICD‐10. The ICD‐10 system is hierarchical, with diseases grouped into chapters based on body systems or disorder types. Each chapter is divided into blocks of related conditions, which in turn contain specific diagnostic codes. The mental and behavioral disorders chapter in the ICD‐10 (F00–F99) contains 11 blocks, with names adjusted in this article for readability and alignment with contemporary terminology. The blocks within mental and behavioral disorders and their definitions can be found in the Appendix S1. Participants were recorded as having a diagnosis if at least one specialist care contact registered a diagnosis in the mental and behavioral disorders blocks during the study period. To test for potential reverse causation, a time‐lagged sensitivity analysis was performed in which exposure diagnoses were restricted to ages 16–17 and outcomes were restricted to ages 18–20.

### 2.2 Outcome metrics

The primary outcome measures were planned visits to neurology clinics, all‐cause and epilepsy‐related emergency department visits, all‐cause and epilepsy‐related unplanned hospital admissions, and mortality. Epilepsy‐related was defined as a registered primary diagnosis of epilepsy (ICD‐10 code G40), status epilepticus (ICD‐10 code G41), or convulsions (ICD‐10 code R56). The participants who had died by the time of data retrieval on January 1, 2023, were classified as deceased. The YPE included in the study were 21–26 years of age at the time of data retrieval. All outcome measures were dichotomous (yes/no) and reflected whether the outcome had occurred during the study period, irrespective of the number of events. Age was calculated as the difference between the current calendar year and the participant's birth year, thus representing the age attained during the current year.

### 2.3 Ethical considerations

Ethical approval for this study was obtained from the Swedish Ethical Review Authority (Dnr 2021.06031–01). Data were pseudonymized before access was given.

### 2.4 Statistical analysis

Participants were categorized based on whether they had any psychiatric disorders (ICD‐10 F00–F99, excluding F70–F79) or intellectual disability (ID) (ICD‐10 F70–F79). We also examined each of the 10 psychiatric disorder groups separately as exposures for each outcome. The presence of other comorbidity was adjusted in a second analysis to test whether significant associations were attributable to differences in somatic comorbidity. Adjustment was performed by identifying individuals with any diagnosis within the relevant ICD‐10 chapters in the NPR, which was then included as a covariate. When adjusting for diseases of the nervous system (ICD‐10 code G00–G99), epilepsy and status epilepticus were excluded (G40, G41). Additional possible confounders adjusted for in the secondary analysis were sex, family income, immigration status, and presence of secondary/tertiary hospital in municipality and region. These covariates were included to assess whether observed differences in healthcare utilization could be explained by somatic comorbidity, sociodemographic factors, or variation in healthcare access rather than psychiatric comorbidity alone.

Statistical analyses were performed using SPSS version 29 (IBM SPSS Statistics, IBM Corporation). Group comparisons for the effect of the presence of psychiatric and intellectual disorders on each outcome were conducted using chi‐squared tests. The association between individual ICD‐10 blocks within psychiatric disorders and the chosen outcome measures was assessed using binary logistic regression. Analyses adjusting for somatic comorbidity, sex, family income, and immigration status were also performed using binary logistic regression. Statistical tests were two‐sided, and two‐sided *p*‐values below 0.05 were considered significant. We did not adjust *p*‐values for multiple testing, and subgroup findings should therefore be interpreted as exploratory and hypothesis‐generating. Multicollinearity was assessed using variance inflation factors, all of which were below two, indicating no evidence of problematic multicollinearity.

When performing binary logistic regression, variables that were hypothesized to affect the outcome measures (psychiatric and intellectual disorders and subgroups within psychiatric disorders) were entered first, and when adjusting for confounders, these were entered in a second step using Forward Wald, a stepwise method to include variables found to improve the model. Analyses were limited to variables for which both outcome groups (“no” and “yes”) included at least five cases. Results are presented using odds ratios (OR), adjusted odds ratios (aOR), and 95% confidence intervals (CI). Model performance was assessed using the Hosmer‐Lemeshow test and receiver operating characteristic (ROC) curve analysis and ROC area under the curve (AUC).

## 3 RESULTS

### 3.1 Demographics

A total of 2116 individuals met the criteria for inclusion in the study, out of 542 389 adolescents who were 18 years old during 2015–2019. Of the 2116, 48 were deceased at the time of data retrieval in 2023.

Among the 2116 individuals included, 1195 (56.5%) were diagnosed with a mental or behavioral disorder between the ages of 16 and 20 (Figure 1). Among the 11 ICD‐10 blocks within mental and behavioral disorders, the most common were ID (32.9%), disorders of psychological development (24.8%), childhood behavioral disorders (17.8%), neurotic disorders (12.9%), and mood disorders (7.0%). The less common diagnoses were substance use disorders (2.5%), physiological behavioral syndromes (2.5%), personality disorders (1.2%), organic mental disorders (1.1%), schizophrenia (0.8%), and unspecified mental disorder (0.5%) (Table 1).

**FIGURE 1:**
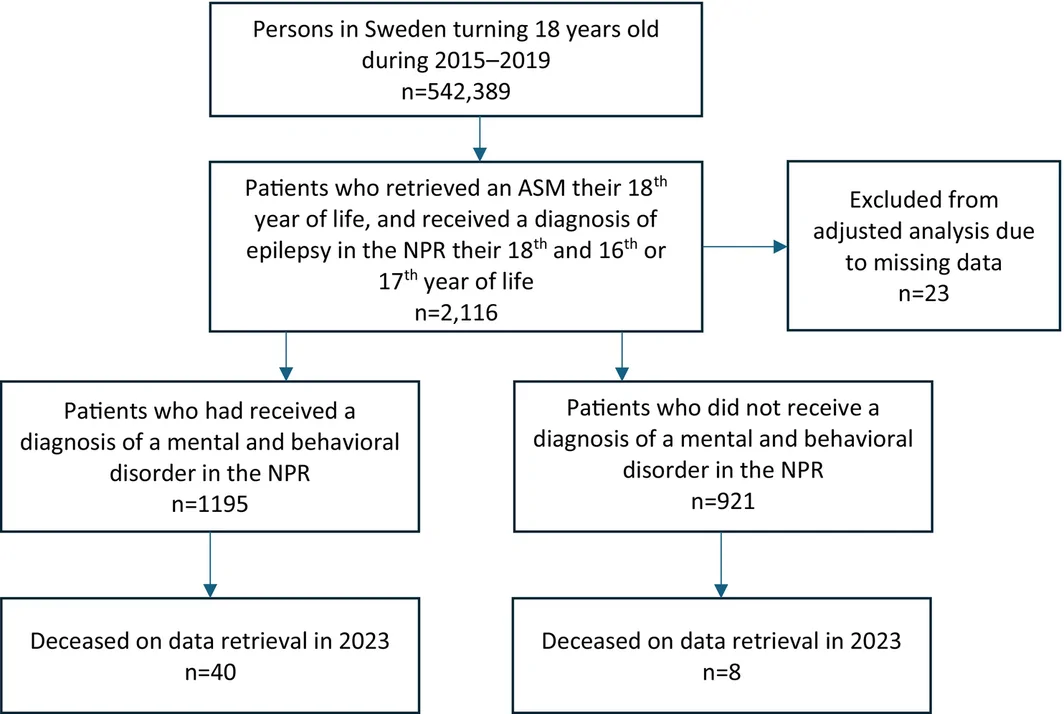
Flow diagram of the study participants. ASM, antiseizure medication; NPR, Swedish National Patient Register.

**TABLE 1:** Clinical characteristics. Number presented first is absolute number, within parenthesis is percentage of total.

Sex and socioeconomics	
Male	1033 (48.8%)
Female	1083 (51.2%)
ASM usage	
Prescribed two or more ASMs for two consecutive years	923 (43.6%)
Prescribed three or more ASMs for two consecutive years	271 (12.8%)
Epilepsy type	
Localization‐related (focal) (partial) idiopathic epilepsy and epileptic syndromes with seizures of localized onset (G40.0)	99 (4.7%)
Localization‐related (focal) (partial) symptomatic epilepsy and epileptic syndromes with simple partial seizures (G40.1)	143 (6.8%)
Localization‐related (focal) (partial) symptomatic epilepsy and epileptic syndromes with complex partial seizures (G40.2)	763 (36.1%)
Generalized idiopathic epilepsy and epileptic syndromes (G40.3)	769 (36.3%)
Other generalized epilepsy and epileptic syndromes (G40.4)	200 (9.5%)
Special epileptic syndromes (G40.5)	14 (0.7%)
Grand mal seizures, unspecified (with or without petit mal) (G40.6)	29 (1.4%)
Petit mal, unspecified, without grand mal seizures (G40.7)	31 (1.5%)
Psychiatric comorbidity	
Mental and behavioral disorders (F00–F99)	1195 (56.5%)
Psychiatric disorders (F00–F99, except F70–F79)	907 (42.9%)
Intellectual disability (F70–F79)	697 (32.9%)
Multiple mental and behavioral disorders across different ICD‐10 blocks	649 (30.7%)
Organic mental disorders (F00–F09)	23 (1.1%)
Substance use disorders (F10–F19)	53 (2.5%)
Schizophrenia (F20–F29)	16 (0.8%)
Mood disorders (F30–F39)	148 (7.0%)
Neurotic disorders (F40–F48)	273 (12.9%)
Psychological behavioral syndromes (F50–F59)	52 (2.5%)
Personality disorders (F60–F69)	25 (1.2%)
Developmental disorders (F80–F89)	525 (24.8%)
Childhood behavioral disorders (F90–F98)	376 (17.8%)
Unspecified mental disorders (F99)	11 (0.5%)
Outcome measures	
Planned visits to neurology clinics	1339 (63.3%)
Emergency care visits	1535 (72.5%)
Unplanned inpatient care	882 (41.7%)
Unplanned inpatient care for epilepsy	354 (22.9%)
Epilepsy‐related emergency care contact	840 (39.7%)
Deceased at data retrieval (2023, age 17–26)	48 (2.3%)

Abbreviation: ASM, antiseizure medication.

The sex distribution was 51.2% female and 48.8% male. Regarding ASM use, 43.6% had been prescribed at least two ASMs for two consecutive years, indicating usage of two concurrent ASMs, while 12.8% had been prescribed at least three ASMs for two consecutive years.

The most common epilepsy type in our study was generalized idiopathic epilepsy and epileptic syndromes (36.3%), with localization‐related (focal) (partial) symptomatic epilepsy and epileptic syndromes with complex partial seizures being the second most common (36.1%).

Overall, 72.5% of participants had at least one all‐cause emergency department visit, and 41.7% had at least one all‐cause unplanned hospital admission. Furthermore, 22.9% had at least one epilepsy‐related unplanned hospital admission, and 39.7% had at least one epilepsy‐related emergency department visit. The mortality was 48/2116 (2.3%) at the time of data retrieval in 2023.

### 3.2 Association of mental and behavioral disorders and epilepsy care outcomes

A diagnosis of a psychiatric disorder was associated with increased odds of all‐cause emergency care (OR 1.59), all‐cause unplanned inpatient care (OR 1.98), and increased mortality (OR 3.95) (Table 2). After adjustment for covariates, the increased odds of all‐cause emergency care (aOR 1.72) and unplanned inpatient care persisted (aOR 1.55), but the higher mortality did not. However, in the adjusted analysis, psychiatric disorders displayed increased odds of unplanned inpatient care for epilepsy (aOR 1.80, CI 1.24–2.61) and epilepsy‐related emergency care contacts (aOR 1.45, CI 1.20–1.74). A psychiatric diagnosis was recorded as the main diagnosis in approximately one‐tenth of emergency visits (11.3%) and inpatient visits (11.8%) among patients with a psychiatric diagnosis.

**TABLE 2:** Associations between psychiatric‐ and intellectual disorders with the six outcome measures.

Variables		Planned visit to neurology clinic	All‐cause emergency care	All‐cause unplanned inpatient care	Epilepsy‐related unplanned inpatient care	Epilepsy‐related emergency care	Deceased upon data retrieval in 2023
Psychiatric disorders (F00–F99, except F70–F79), not adjusted for confounders	Odds ratio, CI	1.05 (0.88–1.26)	1.59 (1.31–1.92)***	1.98 (1.66–2.37)***	1.20 (0.98–1.47)	1.04 (0.87–1.24)	3.95 (1.84–8.49)***
Intellectual disability (F70–F79), not adjusted for confounders	Odds ratio, CI	0.99 (0.82–1.20)	1.07 (0.87–1.31)	1.57 (1.31–1.88)***	1.14 (0.92–1.40)	0.84 (0.70–1.01)	5.72 (3.01–10.88)***
Psychiatric disorders (F00–F99, except F70–F79), adjusted for confounders	Adjusted odds ratio, CI	1.04 (0.86–1.26)	1.72 (1.38–2.15)***	1.55 (1.27–1.88)***	1.80 (1.24–2.61)**	1.45 (1.20–1.74)***	0.85 (0.46–1.58)
Intellectual disability (F70–F79), adjusted for confounders	Adjusted odds ratio, CI	0.94 (0.77–1.15)	0.56 (0.44–0.72)***	0.87 (0.70–1.09)	1.20 (0.88–1.62)	0.72 (0.59–0.88)**	2.57 (1.27–5.20)**

*Note*: Confounders adjusted for were somatic disease, sex, family income, immigration status and presence of secondary/tertiary hospital in municipality and region. Twenty‐three individuals were excluded from the analysis adjusted for confounders due to missing data. Both values unadjusted and adjusted for confounding variables are shown. Statistical significance is indicated by bold values and asterisks, where *p* < 0.05 (*), *p* < 0.01 (**), and *p* < 0.001 (***).

A diagnosis of ID was associated with increased odds of receiving all‐cause unplanned inpatient care (OR 1.57) and increased mortality (OR 5.72). When adjusting for covariates, the increased mortality persisted (aOR 2.57, CI 1.27–5.20), but the increased odds of all‐cause unplanned inpatient care did not. In the adjusted analysis, ID was, however, linked to lower odds of all‐cause emergency care (aOR 0.56, CI 0.44–0.72) and epilepsy‐related emergency care (aOR 0.72, CI 0.59–0.88).

When performing subgroup analysis on the diagnoses within psychiatric disorders and their association with the six outcome measures after adjustment for covariates, none of the variables were found to be associated with planned visits to neurology clinics, epilepsy‐related emergency care contacts, or mortality (Table 3). Higher odds of all‐cause emergency care were found in mood disorders (aOR 2.32), neurotic disorders (aOR 2.14), and childhood behavioral disorders (aOR 1.38). Substance use disorders and neurotic disorders were linked to both increased odds of all‐cause unplanned inpatient care (aOR 2.83; aOR 1.71) and higher odds of epilepsy‐related unplanned inpatient care (aOR 2.20; aOR 1.67). Unadjusted subgroup analysis for psychiatric disorders is available in Table S1.

**TABLE 3:** Associations between the 10 ICD‐10 blocks within psychiatric disorders (F00–F99, except F70–F79) and the 6 outcome measures, adjusted for confounders.

Subcategories within psychiatric disorders	Planned visit to neurology clinic	All‐cause emergency care	All‐cause unplanned inpatient care	Epilepsy‐related unplanned inpatient care	Epilepsy‐related emergency care	Deceased upon data retrieval in 2023
Organic mental disorders (F00–F09)	1.28 (0.50–3.28)			1.96 (0.82–4.67)	2.19 (0.92–5.21)	
Substance use disorders (F10–F19)	1.09 (0.60–1.97)		2.83 (1.45–5.51)**	2.20 (1.22–3.94)*	1.76 (0.99–3.11)	
Schizophrenia (F20–F29)	0.91 (0.30–2.73)			1.93 (0.68–5.51)	1.30 (0.47–3.62)	
Mood disorders (F30–F39)	0.79 (0.53–1.18)	2.32 (1.31–4.12)**	1.51 (0.99–2.29)	0.90 (0.58–1.40)	1.12 (0.76–1.65)	
Neurotic disorders (F40–F48)	1.20 (0.88–1.64)	2.14 (1.40–3.24)***	1.71 (1.24–2.35)***	1.67 (1.22–2.30)**	1.31 (0.98–1.75)	
Physiological behavioral syndromes (F50–F59)	0.85 (0.46–1.57)		1.41 (0.74–2.67)	1.06 (0.55–2.05)	1.00 (0.55–1.82)	
Personality disorders (F60–F69)	1.86 (0.69–4.97)		1.62 (0.62–4.19)	0.39 (0.12–1.27)	1.58 (0.66–3.73)	
Disorders of psychological development (F80–F89)	0.90 (0.73–1.10)	0.83 (0.63–1.10)	1.16 (0.91–1.48)	1.11 (0.86–1.43)	1.19 (0.95–1.50)	0.67 (0.33–1.38)
Childhood behavioral disorders (F90–F98)	1.20 (0.95–1.52)	1.38 (1.02–1.87)*	1.03 (0.79–1.35)	1.09 (0.82–1.44)	1.08 (0.85–1.38)	1.04 (0.41–2.59)
Unspecified mental disorder (F99)				2.91 (0.76–11.19)		

*Note*: Boxes without numbers have been excluded due to too few cases for one or both outcomes. Twenty‐three individuals were excluded from the adjusted analysis due to missing data. Statistical significance is indicated by bold values and by asterisks, where *p* < 0.05 (*), *p* < 0.01 (**), and *p* < 0.001 (***). Adjusted odds ratios are presented in each cell, with 95% confidence intervals shown in parentheses.

The adjusted time‐lagged sensitivity analysis showed persistently increased odds of all‐cause emergency care (aOR = 1.32) and unplanned inpatient care (aOR = 1.53) in young persons with epilepsy and psychiatric disorders. The association with increased epilepsy‐related unplanned inpatient care and emergency care did not persist. ID was linked to lower odds of all‐cause emergency care (aOR = 0.66, CI = 0.53–0.83), but the associations with decreased rates of epilepsy‐related emergency care contacts and increased mortality did not persist (Table 4).

**TABLE 4:** Time‐lagged sensitivity analysis showing associations between psychiatric‐ and intellectual disorders with the six outcome measures.

Variables		Planned visit to neurology clinic	All‐cause emergency care	All‐cause unplanned inpatient care	Epilepsy‐related unplanned inpatient care	Epilepsy‐related emergency care	Deceased upon data retrieval in 2023
Psychiatric disorders (F00–F99, except F70–F79), not adjusted for confounders	Odds ratio, CI	1.20 (0.99–1.45)	1.37 (1.14–1.65)***	1.64 (1.34–2.00)***	1.22 (0.93–1.60)	1.14 (0.94–1.39)	0.70 (0.37–1.34)
Intellectual disability (F70–F79), not adjusted for confounders	Odds ratio, CI	0.99 (0.81–1.20)	1.10 (0.91–1.34)	1.57 (1.28–1.94)***	1.39 (1.06–1.84)*	0.99 (0.80–1.22)	3.03 (1.71–5.39)***
Psychiatric disorders (F00–F99, except F70–F79), adjusted for confounders	Adjusted odds ratio, CI	1.17 (0.95–1.43)	1.32 (1.08–1.62)**	1.53 (1.22–1.92)***	1.16 (0.87–1.54)	1.16 (0.94–1.42)	0.69 (0.35–1.37)
Intellectual disability (F70–F79), adjusted for confounders	Adjusted odds ratio, CI	0.95 (0.77–1.18)	0.66 (0.53–0.83)***	0.87 (0.67–1.11)	1.19 (0.89–1.60)	0.90 (0.72–1.13)	1.41 (0.74–2.70)

*Note*: Confounders adjusted for were somatic disease, sex, family income, immigration status and presence of secondary/tertiary hospital in municipality and region. Twenty‐three individuals were excluded from the analysis adjusted for confounders due to missing data. Shown both unadjusted and adjusted for confounding variables. Statistical significance is indicated by bold values and asterisks, where *p* < 0.05 (*), *p* < 0.01 (**), and *p* < 0.001 (***).

## 4 DISCUSSION

In this population‐based nationwide study, we found associations between psychiatric comorbidities and ID with healthcare utilization and mortality in young people with epilepsy. Overall, over 70% of YPE had unplanned emergency visits in the 5‐year span of reaching adulthood, a clear illustration of adolescence and transition as a vulnerable time for PWE. YPE with psychiatric comorbidities had increased odds of all‐cause and epilepsy‐related emergency visits and hospital admissions. The findings for epilepsy‐related care did not persist in the time‐lagged sensitivity analysis, which may suggest that epilepsy severity and psychiatric diagnoses co‐occur, or that poorer seizure control may precede psychiatric diagnoses. The YPE with ID had an increased mortality, even after adjusting for confounding somatic diseases. In addition, YPE with ID had more frequent all‐cause inpatient care, but this association did not persist when adjusting for covariates.

Our findings regarding increased mortality among persons with ID and psychiatric comorbidity are consistent with findings from prior studies.^27^, ^31^ When adjusting for covariates, this association disappeared for psychiatric disorders, but was still present for ID in the main adjusted analysis. In PWE with ID, this increase is partially explained by associated somatic comorbidities such as infectious diseases, metabolic diseases, and nervous system diseases, which are found in the current study and other studies.^4^ However, the increased mortality persisted in YPE with ID after adjustment for confounders in our cohort, consistent with a study showing increased mortality among teenagers with epilepsy and ID after adjustment for somatic comorbidity and socioeconomic factors.^32^ A possible explanation might be that epilepsy is a marker of the severity of the disability/neurological disease, and this severity is a predictor for mortality.^33^ Previous studies suggest that the association to increased mortality may be stronger in females and at younger ages, and the association decreased with age, possibly reflecting a “healthy survivor effect”.^32^ For example, individuals with epilepsy and comorbid ID are reported to have an elevated risk of death from external causes, including accidents and suicide.^28^

The association between psychiatric comorbidity and increased emergency department visits and unplanned hospital admissions supports prior work regarding adults with psychiatric comorbidity.^23^ The study by Sajatovic et al.^23^ did not specify the cause of the emergency department visit, while a study by Chuah et al.^24^ showed a specific increase of epilepsy‐related visits. Chuah et al. also found a decreased level of follow‐up in persons with psychiatric comorbidity. This, in combination with the association of increased mortality in PWE and psychiatric comorbidity,^27^, ^28^ underlines the importance of more outpatient follow‐up opportunities, specifically for people with epilepsy and comorbid psychiatric disorders. Among individuals with psychiatric disorders, approximately one‐tenth of emergency department visits and inpatient admissions had a main diagnosis of a mental or behavioral disorder. This suggests that the increased healthcare utilization in this group was not solely driven by psychiatric presentations, and that psychiatric comorbidity may also influence somatic healthcare utilization patterns. The decreased odds of all‐cause and epilepsy‐related emergency visits among YPE with ID in the main adjusted analysis were unexpected and contrary to an earlier analysis showing increased emergency care among YPE with comorbid ID.^25^ However, this study did not account for somatic comorbidity, which is more common among persons with ID.^15^ The presence of ID may reduce the odds of emergency care, possibly through a different standard of care or caregiver management of crises. However, YPE with ID still suffer a much higher risk of mortality, and continued efforts to improve their care are therefore warranted. This should also be interpreted in light of the close relationship between ID and psychiatric comorbidity, as psychiatric disorders are more common and may present differently in individuals with ID.^34^ Although interaction analyses were beyond the scope of the present study, co‐occurring ID and psychiatric disorders may represent a subgroup of YPE with particularly complex care needs and should be examined in future studies.

In specific psychiatric disorders, several diagnoses were associated with increased healthcare utilization. For example, substance use disorders showed increased all‐cause and epilepsy‐related unplanned inpatient care, consistent with earlier studies showing that, for example, substance abuse in adults may increase seizure frequency and severity while worsening medication adherence.^35^ Mood disorders were associated with a higher risk of all‐cause emergency care, consistent with earlier findings.^25^ Neurotic disorders showed increased odds of all‐cause emergency care and unplanned inpatient care, as well as epilepsy‐related unplanned inpatient care. Earlier studies have shown that the presence of neurotic disorders might decrease response to initial treatment with ASMs and increase the risk of seizure recurrence,^36^, ^37^ and have found increased emergency visits in multivariate analysis. The presence of childhood behavioral disorders was associated with higher odds of all‐cause emergency visits, consistent with earlier studies showing increased emergency department visits among YPE with comorbid attention deficit hyperactivity disorder (ADHD).^25^ Multiple diagnoses within psychiatric disorders were associated with increased healthcare utilization and mortality, but most associations disappeared in the adjusted analysis, indicating confounding by the included covariates. In conclusion, under‐diagnosis and under‐treatment of psychiatric comorbidities may lead to worse seizure control and reduced life expectancy.

Multipsychiatric morbidity in people with epilepsy was especially common, with 30.7% having at least two different psychiatric diagnoses within different blocks within mental and behavioral disorders. Earlier studies have shown increased multipsychiatric morbidity among people with epilepsy compared to people without; however, these studies looked at specific combinations of multipsychiatric morbidity, and not the prevalence of overall multipsychiatric morbidity.^38^

### 4.1 Strengths and limitations

The main strengths of this register‐based study lie in the comprehensive national healthcare coverage and high data credibility with low dropout rates.^39^ To increase the probability of participants having epilepsy we required at least two codings and their validity was supported by ASM prescriptions. The time‐lagged sensitivity analysis strengthens the temporal ordering between exposure and outcome, but its shorter ascertainment window increases the risk of under‐identifying chronic psychiatric disorders. The results of the study are limited by missing data from primary care. For example, depression and anxiety are often managed in primary care in Sweden and our prevalence of mood and neurotic disorders was lower than previously reported. Moreover, ICD‐10 diagnoses recorded for administrative purposes often have low sensitivity for psychiatric disorders as many true cases are never recorded.^40^ Furthermore, as in all registry studies, limitations related to data quality and scope exist. For example, we were unable to assess seizure frequency or ASM adherence.

### 4.2 Clinical implications and future research

The high prevalence of psychiatric comorbidities and ID among adolescents with epilepsy, and their association with increased healthcare utilization and mortality, underscore the need for integrated care models for adolescents, especially during transition from child to adult healthcare. Screening and assessment for psychiatric and intellectual disorders should be systematically incorporated into routine epilepsy care during adolescence. Early identification of comorbidities may enable tailored interventions aimed at reducing emergency department visits, unplanned hospitalizations, and adverse outcomes.

The differing healthcare utilization patterns observed for psychiatric comorbidities and ID suggest that strategies during transition should be individualized. Adolescents with psychiatric comorbidities without ID may benefit from strengthened outpatient follow‐up, crisis prevention plans, and closer collaboration between neurology and mental health services. For those with ID and/or ID and psychiatric disorders, structured care coordination and caregiver‐supported transition planning may be particularly important to reduce inpatient admissions and ensure continuity of care. From a clinical perspective, these findings highlight the need for integrated care pathways and agreed standards of care across neurology and psychiatry services, pediatric and adult healthcare, and community and hospital settings. Developing coordinated service models in partnership with young people, families, and carers may help reduce fragmentation and improve the quality and responsiveness of care during the transition to adulthood.

Future research should explore the mechanisms underlying the increased mortality observed in this population, including the potential roles of treatment adherence, socioeconomic factors, and healthcare accessibility. Longitudinal studies examining patient‐reported outcomes, quality of life, and engagement with adult care services would further clarify how comorbidities influence long‐term trajectories. Additionally, interventional studies should aim to improve care pathways and transition programs between pediatric and adult clinics, between neurology and psychiatry clinics, and between hospital care, primary care, and social care to improve outcomes for young people with epilepsy. Additionally, to ensure optimal outcomes, research efforts should be developed in partnership with adolescents, caregivers, and healthcare staff.

## 5 CONCLUSION

A high prevalence of psychiatric comorbidities and ID was found in YPE, and these comorbidities were associated with increased mortality and acute healthcare consumption. YPE with psychiatric comorbidities had increased odds of all‐cause and epilepsy‐related emergency department visits or inpatient admissions. YPE with ID had a different pattern, with fewer emergency department visits but higher mortality. These findings highlight the increased vulnerability among YPE with psychiatric comorbidities and ID and may help develop effective interventions for care transitions and long‐term outcomes in adolescents with epilepsy. These results also emphasize the importance of individualized care planning and comprehensive assessments for young people with epilepsy, particularly those with psychiatric and intellectual comorbidities.

## AUTHOR CONTRIBUTIONS

**Jesse Sobovitch**: Writing original draft, data and statistical analysis. **Colin Reilly**: Data validation, manuscript review and editing, and subject matter expertise. **Johan Zelano**: Manuscript review and editing, and subject‐matter expertise. **Rianne Goselink**: Project administration, data acquisition, study design, supervision, and manuscript review and editing.

## FUNDING INFORMATION

The work was supported by Epilepsiförbundet Sverige, Linnéa och Josef Carlssons Stiftelse, and Margarethahemmet.

## CONFLICT OF INTEREST STATEMENT

Rianne Goselink, Colin Reilly, and Jesse Sobovitch have no conflicts of interest to disclose. Johan Zelano reports speaker/advisory board honoraria from UCB, Eisai, Orion Pharma, Angelini Pharma, and Sanofi, and as an employee of Sahlgrenska University Hospital, being an investigator in clinical trials sponsored by GW Pharma, SK Life science, Bial, UCB, and Angelini Pharma (no personal compensation).

## ETHICAL PUBLICATION STATEMENT

We confirm that we have read the Journal's position on issues involved in ethical publication and affirm that this report is consistent with those guidelines.

## Supporting information


Table S1.

## Data Availability

The data that support the findings of this study are available on request from the corresponding author. The data are not publicly available due to privacy or ethical restrictions.
