# Supplementary material for: Psychiatric disorders and intellectual disability impact epilepsy care in adolescents: A nationwide registry study
Source: Epilepsia Open. 2026 Aug 1;11(5):1603–14. doi: 10.1002/epi4.70314 (PMC13428350; doi:10.1002/epi4.70314)
Supplement: Supplementary file 1 [file EPI4-11-1603-s001.docx]

**Table S1**

| Associations between the ten ICD-10 blocks within psychiatric disorders (F00-F99, except F70-F79) and the six outcome measures, not adjusted for confounders. Statistical significance is indicated by bold text and by asterisks, where p < 0.05 (*), p < 0.01 (**), and p < 0.001 (***). Odds ratios are presented in each cell, with 95% confidence intervals shown in parentheses. | | | | | | |
| --- | --- | --- | --- | --- | --- | --- |
| Mental and behavioral disorders | Planned visit to neurology clinic | All-cause emergency care | All-cause unplanned inpatient care | Epilepsy-related unplanned inpatient care | Epilepsy-related emergency care | Deceased upon data retrieval in 2023 |
| Organic mental disorders (F00-F09) | 1.33 (0.55–3.25) |  |  | **2.51 (1.09–5.76)*** | **2.39 (1.03–5.54)*** |  |
| Substance use disorders (F10-F19) | 1.04 (0.59–1.84) |  | **3.66 (2.00–6.69)***** | **2.54 (1.46–4.42)***** | **2.02 (1.16–3.50)*** |  |
| Schizophrenia (F20-F29) | 1.28 (0.44–3.70) |  |  | 2.53 (0.94–6.82) | 1.96 (0.73–5.29) |  |
| Mood disorders (F30-F39 | 0.89 (0.63–1.26) | **3.32 (1.96–5.63)***** | **2.30 (1.64–3.24)***** | 1.21 (0.83–1.77) | **1.44 (1.03–2.01)*** |  |
| Neurotic disorders (F40-F48) | 1.16 (0.89–1.52) | **2.86 (1.98–4.14)***** | **2.40 (1.85–3.11)***** | **1.78 (1.36–2.34)***** | **1.50 (1.16–1.94)**** |  |
| Physiological behavioral syndromes (F50-F59) | 0.93 (0.53–1.63) |  | **2.10 (1.20–3.69)**** | 1.32 (0.72–2.42) | 1.42 (0.82–2.46) |  |
| Personality disorders (F60-F69) | 1.85 (0.74–4.65) |  | **3.01 (1.29–7.01)**** | 0.80 (0.30–2.15) | **2.30 (1.03–5.15)*** |  |
| Disorders of psychological development (F80-F89) | 1.23 (1.00–1.52) | 1.14 (0.91–1.43) | **1.47 (1.20–1.79)***** | 1.21 (0.96–1.51) | 1.17 (0.95–1.42) | 1.01 (0.52–1.96) |
| Childhood behavioral disorders (F90-F98) | 1.09 (0.86–1.38) | **1.50 (1.15–1.96)**** | **1.27 (1.02–1.59)*** | 1.23 (0.95–1.59) | 1.22 (0.97–1.53) | 0.66 (0.28-1.55) |

Cells without numbers have been excluded due to too few cases for one or both outcomes.

Appendix S1

**Blocks within mental and behavioral disorders (F00-F99):**

**1. Organic mental disorders (F00–F09)**

Disorders caused by brain injury, disease, or dysfunction:

Dementia (Alzheimer’s disease, vascular dementia)

Delirium

Amnestic syndromes

Other organic mental disorders

**2. Substance use disorders (F10–F19)**

Problems related to use of psychoactive substances:

Alcohol use disorder

Opioid use disorder

Cannabis, sedative, stimulant, and other substance-related disorders

Substance-induced mental disorders (e.g., intoxication, withdrawal)

**3. Schizophrenia, schizotypal, delusional, and other psychotic disorders (F20–F29)**

Severe mental disorders affecting thinking and perception:

Schizophrenia

Schizoaffective disorder

Delusional disorder

Brief psychotic disorder

Other nonorganic psychoses

**4. Mood (affective) disorders (F30–F39)**

Disorders affecting mood:

Bipolar disorder (F30–F31)

Major depressive disorder (F32)

Recurrent depressive disorder (F33)

Persistent mood disorders like dysthymia (F34)

Other mood disorders (F38, F39)

**5. Neurotic, stress-related, and somatoform disorders (F40–F48)**

Anxiety and stress-related conditions:

Phobic anxiety disorders (F40)

Other anxiety disorders (F41)

Obsessive-compulsive disorder (F42)

Reaction to severe stress, adjustment disorders (F43)

Dissociative (conversion) disorders (F44)

Somatoform disorders (F45)

Other neurotic disorders (F48)

**6. Behavioral syndromes associated with physiological disturbances (F50–F59)**

Disorders linked to physiological function:

Eating disorders (anorexia nervosa, bulimia nervosa) (F50)

Sleep disorders (F51)

Sexual dysfunction not due to organic causes (F52)

Other behavioral syndromes associated with physiological disturbances

**7. Personality disorders (F60–F69)**

Long-term patterns of maladaptive behavior and personality traits:

Borderline, antisocial, narcissistic, paranoid, schizoid, avoidant, and other personality disorders

Disorders of adult personality and behavior

**8. Intellectual disability (F70–F79)**

Mild, moderate, severe, and profound intellectual disability

Global developmental delay

**9. Disorders of psychological development (F80–F89)**

Conditions usually diagnosed in childhood:

Specific developmental disorders of speech and language (F80)

Specific developmental disorders of scholastic skills (F81)

Autism spectrum disorder (F84)

Other pervasive developmental disorders (F84.1–F84.9)

Other disorders of psychological development (F88)

**10. Childhood behavioral and emotional disorders (F90–F98)**

Behavioral or emotional problems in children and adolescents:

ADHD (F90)

Conduct disorder (F91)

Emotional disorders with onset in childhood (F92)

Mixed disorders of conduct and emotions (F92.8)

Tic disorders (F95)

Other behavioral/emotional disorders (F93, F98)

**11. Unspecified mental disorder (F99)**

Mental disorder not otherwise specified or unspecified

Used when the diagnosis doesn’t fit other categories
